# Integrating 16S rDNA and metabolomics to uncover the therapeutic mechanism of electroacupuncture in type 2 diabetic rats

**DOI:** 10.3389/fmicb.2024.1436911

**Published:** 2025-01-06

**Authors:** Zhang Yue, Wang Xiang, Deng Duping, Gong Yuanyuan, Chen Xuanyi, Li Juan, Hong Xiaojuan

**Affiliations:** ^1^School of Health Preservation and Rehabilitation, Chengdu University of Traditional Chinese Medicine, Chengdu, China; ^2^School of Acupuncture-Moxibustion and Tuina, Chengdu University of Traditional Chinese Medicine, Chengdu, China; ^3^Department of Rehabilitation Medicine, Meishan Hospital of Traditional Chinese Medicine, Meishan, China; ^4^Affiliated Sichuan Provincial Rehabilitation Hospital of Chengdu University of Traditional Chinese Medicine, Chengdu, China

**Keywords:** electroacupuncture, T2DM, gut microbiota, SCFAs, GLP-1

## Abstract

**Objective:**

This study aimed to investigate the impact of electroacupuncture (EA) on blood glucose levels, gut microbiota, short-chain fatty acids (SCFAs), and glucagon-like peptide-1 (GLP-1) in a rat model of type 2 diabetes mellitus (T2DM).

**Methods:**

Forty Sprague–Dawley (SD) rats were randomly assigned to five groups (n = 8/group) using a random number table: normal control, T2DM model, electroacupuncture (EA), EA + antibiotics (EA + A), and antibiotics (A). The normal rats received a standard diet and saline gavage, while the other groups were fed a high-fat diet and emulsion. The EA + A and A groups received additional antibiotic solution gavage. The normal, model, and A groups were immobilized and restrained for 30 min, six times per week, for 4 weeks. The EA and EA + A groups received EA treatment at specific acupoints for 30 min, six times per week, for 4 weeks. EA parameters were continuous waves at 10 Hz and 1–2 mA. During the intervention, water and food consumption, body weight, fasting blood glucose (FBG), and oral glucose tolerance test (OGTT) were monitored. Pancreatic tissue was examined using hematoxylin and eosin (H&E) staining. Fecal microbial communities were analyzed by 16S rDNA sequencing, and short-chain fatty acids (SCFAs) were measured using gas chromatography–mass spectrometry (GC–MS). Serum levels of fasting insulin (FINS), glycated hemoglobin (HbA1c), and glucagon-like peptide-1 (GLP-1) were determined using enzyme-linked immunosorbent assay (ELISA).

**Results:**

EA significantly improved daily water intake, food consumption, and body weight in T2DM rats (*p* < 0.01). EA also reduced FBG, the area under the curve of the OGTT, FINS, and homeostasis model assessment of insulin resistance (HOMA-IR) in T2DM rats (*p* < 0.05). The ELISA results showed a lower concentration of HbA1c in the EA group (*p* < 0.05). EA improved the overall morphology and area of pancreatic islets, increased the number of *β*-cell nuclei, and alleviated β-cell hypertrophy. The abundance of operational taxonomic units (OTUs) in the EA group increased than the model group (*p* < 0.05), and EA upregulated the Shannon, Chao1, and Ace indices (*p* < 0.05). EA increased the concentrations of acetic acid, butyric acid, and GLP-1 (*p* < 0.05). Correlation analysis revealed negative associations between *Lactobacillaceae* (*R* = −0.81, *p* = 0.015) and *Lactobacillus* (*R* = −0.759, *p* = 0.029) with FBG. *Peptostreptococcaceae* and *Romboutsia* were negatively correlated with HbA1c (*R* = −0.81, *p* = 0.015), while *Enterobacteriaceae* was positively correlated with OGTT (*R* = 0.762, *p* = 0.028). GLP-1 was positively correlated with acetic acid (*R* = 0.487, *p* = 0.001), butyric acid (*R* = 0.586, *p* = 0.000), isovaleric acid (*R* = 0.374, *p* = 0.017), valeric acid (*R* = 0.535, *p* = 0.000), and caproic acid (*R* = 0.371, *p* = 0.018). Antibiotics disrupted the intestinal microbiota structure and weakened the therapeutic effects of EA.

**Conclusion:**

EA effectively improved glucose metabolism in T2DM rats. The hypoglycemic effects of EA were associated with the regulation of gut microbiota, SCFAs, and GLP-1.

## 1 Introduction

Diabetes is characterized by hyperglycemia, resulting from insulin resistance (IR) and impaired insulin secretion by pancreatic beta cells. Due to unhealthy lifestyles and accelerated aging, the number of individuals with diabetes is increasing globally. According to the International Diabetes Federation, in 2021, 537 million adults (20–79 years old) worldwide had diabetes (10.5% prevalence), and this number is projected to rise to 643 million (11.3%) by 2030 and 783 million (12.2%) by 2045 (Sun et al., 2022). Among these, 90% of cases are type 2 diabetes mellitus (T2DM). In 2021, global diabetes-related healthcare expenditures reached US$966 billion (Sun et al., 2022). In China, age-standardized incidence, prevalence, and mortality rates for T2DM are 4,496.4/100,000, 201.1/100,000, and 9.2/100,000, respectively (Cheng et al., 2023). Currently, the primary clinical treatment for T2DM relies on oral hypoglycemic drugs and insulin injections (American Diabetes Association Professional Practice Committee, 2024). However, the efficacy of oral hypoglycemic drugs is limited due to drug tolerance (Díaz-Perdigones et al., 2022; Foretz et al., 2023), hepatic and renal toxicity (Kohler et al., 2017), and gastrointestinal side effects (Tong et al., 2023). Insulin injections are associated with hypoglycemia and weight gain (Satirapoj et al., 2020). Therefore, non-pharmacological therapies have received more attention.

Acupuncture has been shown to lower blood glucose levels, improve IR, reduce the need for hypoglycemic drugs/insulin, and alleviate adverse drug reactions. There is an increasing consensus suggesting that acupuncture can reduce blood glucose levels in T2DM by improving insulin sensitivity, ameliorating insulin resistance, inhibiting pancreatic *β*-cell apoptosis, and modulating insulin signaling pathways (Yingjie et al., 2020; Zhuang et al., 2022; Xu et al., 2022; Zhang et al., 2024). Meta-analyses have also demonstrated the effectiveness of acupuncture in reducing FBG and improving IR (Li et al., 2022). In addition, acupuncture can be used as an adjunctive therapy for hypoglycemic agents (Juntao et al., 2023). However, the hypoglycemic mechanism of EA remains unclear.

The intestinal microbiota plays a crucial role in various physiological processes, including metabolism, energy homeostasis, and immunity (Sanna et al., 2019; Zhernakova et al., 2016). Recent studies have demonstrated a strong association between the intestinal microbiota and the development and progression of T2DM (Sanna et al., 2019; Que et al., 2021). Clinical studies have shown that *Lactobacillus* is linked to low-density lipoprotein cholesterol in individuals with T2DM, and dietary interventions to modulate the intestinal *Lactobacillus* population can benefit diabetes, hyperlipidemia, and other metabolic disorders (Tingting and Dehong, 2012). Tong (2022). reported that fecal microbiota transplantation from T2DM mice increased the relative abundance of *Bifidobacterium*, *Phascolarctobacterium*, and *Collisella* while decreasing the relative abundance of *Muribaculum*, *Ruminiclostridium_5*, and *Lachnospiraceae_FCS020_group*. These changes were associated with reduced FBG and FINS, suggesting that the intestinal microbiota may be a potential therapeutic target for the prevention and treatment of T2DM.

Short-chain fatty acids (SCFAs) are the end products of dietary fiber and host glycan fermentation by the gut microbiota, essential for host physiology and health. Notably, a decrease in butyrate-producing bacteria is strongly associated with insulin resistance and the development of T2DM. As an important incretin, glucagon-like peptide-1 (GLP-1) regulates insulin secretion in a glucose-dependent manner, effectively reducing blood glucose and improving glucose metabolism disorders (Costes et al., 2021). GLP-1 receptor agonists have emerged as important drugs for managing obesity and T2DM (American Diabetes Association Professional Practice Committee, 2024). In addition, SCFAs combine with G protein-coupled receptors to promote GLP-1 secretion, which directly acts on pancreatic islet cells to maintain blood glucose stability (Tolhurst et al., 2012). SCFA supplementation has been reported to affect the satiety response mediated by GLP-1 and peptide YY (PYY) and promote insulin secretion, contributing to glucose homeostasis maintenance (Tolhurst et al., 2012; Christiansen et al., 2018). Zhao et al. found that diets rich in dietary fiber modulated the gut microbiota of T2DM patients, promoted butyric acid production, increased GLP-1 secretion, and ultimately improved glycated hemoglobin (Zhao et al., 2018). Probiotics increased SCFA-producing bacteria and SCFA levels, which enhanced insulin secretion through glucose-triggered GLP-1 secretion by upregulating G protein-coupled receptor 43/41 (GPR43/41), proglucagon, and proconvertase 1/3 activity (Wang et al., 2020). Han et al. documented that fecal microbiota transplantation (FMT) intervention increased the relative abundance of *Bacteroides uniformis* and *Clostridium*, decreased *Mucispirillum schaedleri* levels, and increased acetate and butyrate levels, GPR43 mRNA expression, and GLP-1 protein expression (Han et al., 2021). In summary, the intestinal microbiota metabolite SCFAs play a crucial role in connecting the gut microbiota to GLP-1.

Electroacupuncture (EA) has been shown to ameliorate metabolic disorders in obese rats by increasing beneficial bacteria and reducing harmful bacteria (Ding et al., 2023). Wang et al. (2022) observed that EA had a hypoglycemic effect and increased the abundance of *Firmicutes* and the ratio of *Firmicutes* to *Bacteroidetes* while decreasing the abundance of *Bacteroidetes* and *Eubacterium*. However, the mechanisms by which acupuncture affects SCFAs, regulates gut microbiota, affects SCFAs, stimulates GLP-1 secretion, and exerts a hypoglycemic effect remain unclear. Therefore, we hypothesized that EA reduces blood glucose by influencing gut microbiota, SCFAs, and GLP-1 in T2DM rats. To clarify the crucial role of gut microbiota and SCFAs in the hypoglycemic effect of EA treatment of T2DM, a pseudo-sterile model was established using antibiotic feeding.

## 2 Materials and methods

### 2.1 Reagents and consumables

Stainless acupuncture needles (25 mm × 0.25 mm) were purchased from Suzhou Medical Supplies Factory Co., Ltd., China. EA apparatuses (HANS-200) were obtained from Nanjing Jisheng Medical Technology Co., Ltd., China. A blood glucose meter and blood glucose test paper were provided by Sinocare Company, China. Cholesterol, sodium deoxycholate, Tween-80, propylene glycol, and sucrose were obtained from Beijing Solarbio Science & Technology Co., Ltd., and lard was mixed to prepare a high-fat emulsion. Citric acid and sodium citrate were purchased from Sigma-Aldrich, China. Streptomycin sulfate and penicillin were obtained from Shanghai Aladdin Biochemical Technology Co., Ltd. High-fat feeds were purchased from Chengdu Dossy Experimental Animal Co., Ltd. The enzyme-linked immunosorbent assay (ELISA) kits for fasting insulin (FINS), glycated hemoglobin (HbA1c), and GLP-1 were supplied by MEIMIAN, Jiangsu Feiya Biological Technology Co., Ltd.

### 2.2 Animal model

Forty male Sprague–Dawley (SD) rats, aged 8 weeks and weighing 200 ± 20 g, were purchased from Beijing Si Pei Fu Laboratory Animal Co., Ltd. (Production License: SCXK(Jing)2019–0008). All animal procedures were approved and supervised by the Animal Ethics Committee of Chengdu Senwell Experimental Animals Co., Ltd. (License No. DOSSYLL20220420001). The entire experimental process adhered to the Guide for the Care and Use of Laboratory Animals (8th Edition) published by the U.S. National Institutes of Health (NIH) (National Research Council Committee, 2011) and the Guideline for the Chinese Specifications in the Care and Use of Laboratory Animals. All animals were housed under controlled conditions: temperature (23 ± 1°C), relative humidity 50–60%, and a 12-h light/12-h dark cycle. Food and water were provided *ad libitum*.

After 1 week of adaptive feeding, the rats were randomly assigned to two groups: a normal group (*n* = 8) and a model group (*n* = 32) using a random number table. The normal group was fed a standard diet and received 3 mL/day of 0.9% sodium chloride solution. The model group was fed a high-fat diet and received 3 mL/day of a high-fat emulsion, 6 days/week for 4 weeks. In addition, the model group was intraperitoneally injected with 2% streptozotocin (STZ) at a dose of 30 mg/kg (Manaer et al., 2015). Seventy-two hours post-STZ injection, random blood glucose levels were measured from tail-tip blood samples, and an oral glucose tolerance test (OGTT) was performed. Three days later, random blood glucose levels were measured again. The model of T2DM was considered successfully established if both the previous two random blood glucose levels exceeded 16.7 mmol/L (Zhou et al., 2023) and the 2-h OGTT blood glucose level exceeded 11.1 mmol/L.

### 2.3 Animal grouping and intervention

Eight male SD rats were designated as the normal control group. T2DM model rats were randomly assigned to four groups (*n* = 8/group): model, electroacupuncture (EA), EA combined with antibiotic (EA + A), and antibiotic (A) groups, using a random number table. The rats in the EA + A and A groups received daily oral gavage of 2 mL antibiotic solution (containing 4.0 mg/mL streptomycin and 2.0 mg/mL penicillin) and continued to have *ad libitum* access to the antibiotic solution.

We selected bilateral acupoints Zusanli (ST36), Sanyinjiao (SP6), and Weiwanxiashu (EX-B3). Acupoint localization followed established methods (WHO Regional Office for the Western Pacific, 2022; Medicine NAoTC, 2021): Zusanli, 0.3 cm below the fibular head at the muscle gap, posterior lateral to the knee joint; Sanyinjiao, 10 mm superior to the medial malleolus tip; Weiwanxiashu, 1.5 inches lateral to the lower spinous process of the 8th thoracic vertebra. Disposable stainless-steel needles were inserted 2 mm deep and connected to an EA apparatus (continuous wave, 10 Hz, 1–2 mA). EA and EA + A groups received 30-min EA treatment six times weekly for 4 weeks. The control (normal, model, and A) groups underwent 30-min restraint with the same frequency and duration. Before and after the intervention, we measured FBG and body weight of rats in all groups. In addition, we monitored daily water and food intake.

### 2.4 Sample collection

Blood was collected from the abdominal aorta and left at room temperature for 2 h. Then, the blood was centrifuged (3,000 rpm, 4°C, 15 min), and the serum was carefully collected and kept in liquid nitrogen and stored at −80°C for further analysis. Alcohol was used to sterilize the perianal area of the rats and press the abdomen, and fresh fecal samples were collected into sterile tubes. After collection, the samples were frozen in liquid nitrogen and stored at −80°C. Pancreatic tissue was fixed and preserved using a 4% paraformaldehyde solution for hematoxylin and eosin (HE) staining.

### 2.5 OGTT measurement and area under the curve calculation

After an 8-h fast, FBG levels were measured in rats. Subsequently, they received a 20% glucose solution (1 mL/100 g body weight) via oral gavage. Blood glucose levels were then measured and recorded at 30, 60, 90, and 120 min post-gavage, and the AUC of the blood glucose response was calculated.

### 2.6 FINS, HbA1c, GLP-1 measurement, and homeostasis model assessment-insulin resistance calculation

The concentrations of FINS, HbA1c, and GLP-1 were determined using an ELISA kit according to the manufacturer’s protocol. Absorbance (OD) values were measured at 450 nm and plotted against standard curves to obtain analyte concentrations. Following the determination of FINS concentration, HOMA-IR was calculated using the formula (FBG × FINS)/22.5.

### 2.7 HE staining

The pancreatic tissue was fixed in 4% paraformaldehyde, dehydrated in graded ethanol, embedded in paraffin, sectioned at 4 μm, deparaffinized in xylene and graded ethanol, stained with hematoxylin for 3 min, differentiated, washed, counterstained with eosin for 2 min, dehydrated, cleared, mounted with neutral gum, and examined microscopically (Nikon, Tokyo, Japan).

### 2.8 16S rDNA sequencing

DNA library sequencing was performed on the Illumina HiSeq™ 2500/4000 platform by Gene Denovo Biotechnology Co., Ltd., China. Stool DNA was extracted using HiPure Stool DNA Kits, followed by a quality assessment. PCR amplification targeted the V4 region of the 16S rDNA gene using specific primers 341F (5’-CCTACGGGNGGC WGCAG-3′) and 806R (5’-GGACTACHVGGGTATCTAAT-3′). Amplicons were purified using the AxyPrep DNA Gel Extraction Kit and quantified using the ABI StepOnePlus Real-Time PCR System (Life Technologies, Foster City, United States). Purified amplicons were pooled in equimolar and paired-end sequenced (PE250) on an Illumina platform according to the standard protocols. Raw reads were filtered to remove low-quality sequences and spliced to generate high-quality reads. These reads were clustered into operational taxonomic units (OTUs) at a 97% similarity threshold using the UPARSE pipeline (version 9.2.64). Chimeric sequences were removed using UCHIME, and the remaining effective reads were further analyzed. Representative sequences were selected from each OTU based on abundance. Community composition, indicator species, alpha diversity (Chao1, Ace, Simpson, and Shannon indices), and beta diversity (principal coordinate analysis, PCoA) analyses were conducted. The Chao1 and Ace indices estimate richness, while the Shannon and Simpson indices reflect species diversity. Linear discriminant analysis effect size (LEfSe) was used to identify biomarker flora with statistically significant differences in abundance between groups.

### 2.9 Fecal SCFAs measurement by gas chromatography/mass spectrometry

SCFAs, including acetate, propionate, butyrate, isobutyrate, valeric acid, isovaleric acid, and hexanoic acid, were detected using a Thermo Trace 1,300 gas chromatograph-ISQ7000 mass spectrometer (Thermo Fisher Scientific, United States). The GC program began with an initial temperature of 90°C, followed by a ramp to 120°C by 10°C/min, then to 150°C by 5°C/min, and until to 250°C by 25°C/min, sustained for 2 min. Mass spectrometric detection of metabolites was performed in single ion monitoring (SIM) mode with an electron energy of 70 eV. The concentrations of SCFAs were calculated from a standard linear regression curve.

### 2.10 Statistical analysis

All data were expressed as means ± standard deviation (Mean ± SD). Statistical analyses were conducted using SPSS 25.0 for data analysis and GraphPad Prism 8 for figure generation. Paired *t*-tests were used to compare pre-and post-intervention data within each group. One-way analysis of variance (ANOVA) was employed to compare means between multiple groups, followed by LSD *post-hoc* tests to identify specific significant differences. Bioinformatics analysis of 16S rDNA library sequencing data was performed using the online platform Omicsmart.^1^ A *p*-value of less than 0.05 was considered statistically significant.

## 3 Results

### 3.1 Effect of EA on body weight and consumption of water and diet in T2DM rats

**Figure 1:**
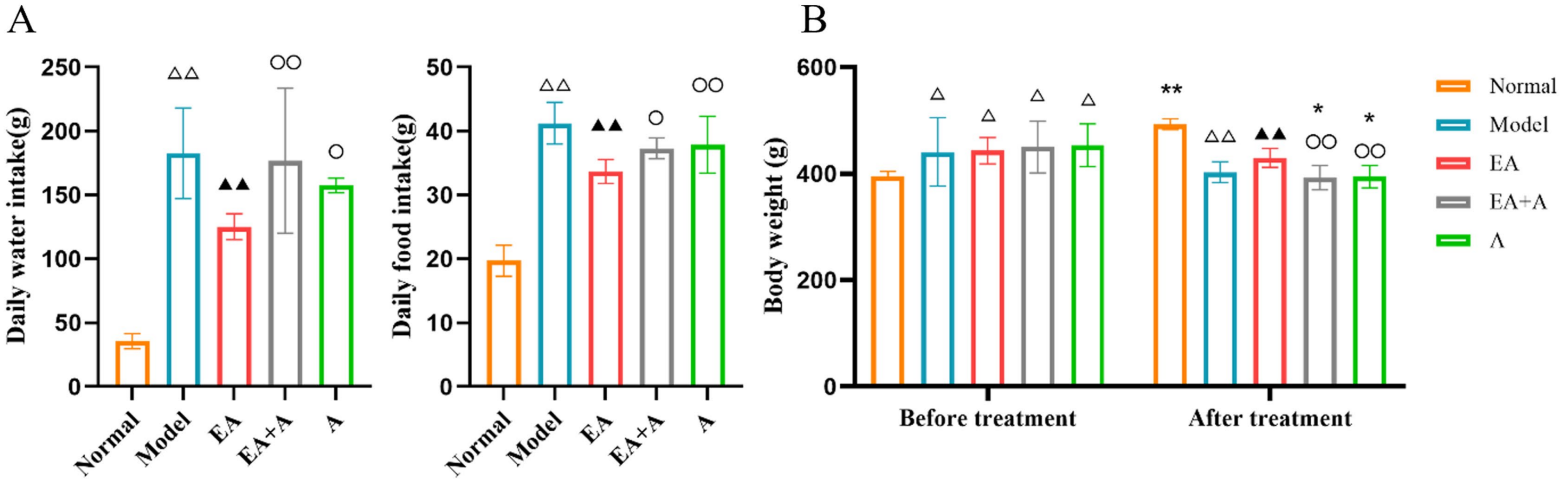
Effect of EA on the body weight and consumption of water and diet. (**A**: Daily water/food intake; **B**: body weight.) **p < 0.01, *p < 0.05, vs. before treatment; ^△△^*p* < 0.01, vs. normal group; ^▲▲^*p* < 0.01, vs. model group; ^○^*p* < 0.05, ^○○^*p* < 0.01, vs. EA group.

Figure 1A illustrates significant alterations in daily water and food consumption across the experimental groups. Compared to the normal group, the model, EA, EA + A, and A groups exhibited substantial increases in both drinking and eating behaviors (*p* < 0.01). Notably, the EA group demonstrated a reduction in consumption relative to the model group (*p* < 0.01). Furthermore, the EA group’s consumption levels were significantly lower than those of the EA + A and A groups (*p* < 0.05).

As shown in Figure 1B, prior to the intervention, the body weight of the model, EA, EA + A, and A groups significantly increased compared to the normal group (*p* < 0.05). However, no significant differences were observed between the model, EA, EA + A, and A groups themselves (*p* > 0.05), suggesting that a high-fat diet is responsible for the elevated body weight in T2DM rats.

Following a 4-week intervention period, a significant difference in body weight was observed among the groups. The normal group exhibited a significant increase in body weight compared to pre-intervention levels (*p* < 0.01). In contrast, the EA + A group and A group experienced significant decreases in body weight compared to pre-intervention levels (*p* < 0.05). Moreover, the EA group demonstrated a significantly higher body weight than the model group (*p* < 0.01). The EA + A group and A group exhibited significantly lower body weights than the EA group (*p* < 0.01).

### 3.2 Effect of EA on the FBG, HbA1c, and the AUC of OGTT of T2DM rats

**Figure 2:**
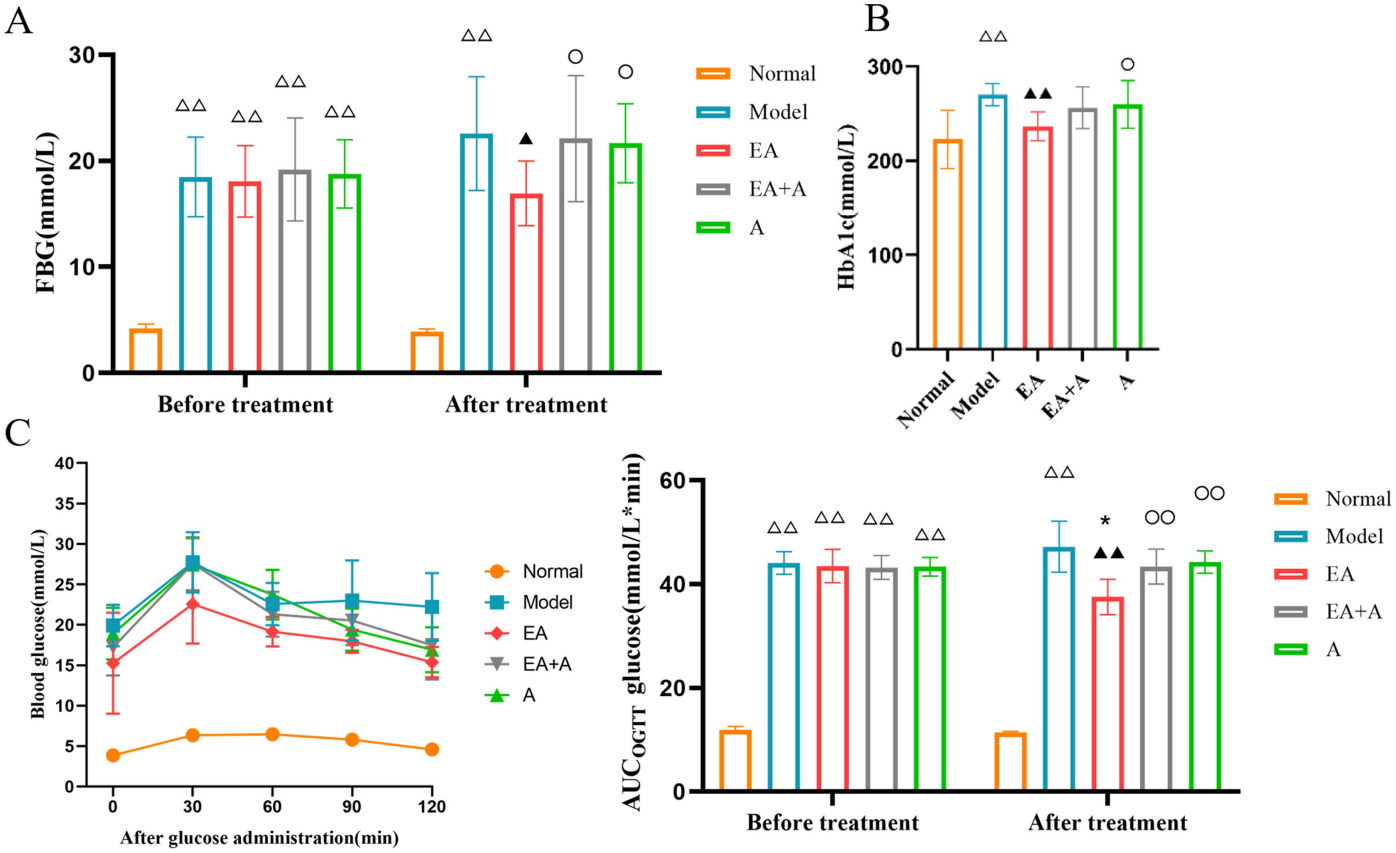
Effect of EA on the FBG, HbA1c, and AUC of OGTT. (**A**: FBG; **B**: HbA1c; **C**: AUC of OGTT.) *p < 0.05, vs. before treatment; ^△△^*p* < 0.01, vs. normal group; ^▲^*p* < 0.05, ^▲▲^*p* < 0.01, vs. model group; ^○^*p* < 0.05, ^○○^*p* < 0.01, vs. EA group.

Figure 2 illustrates the outcomes of FBG (A), HbA1c (B), and OGTT AUC (C). Before the intervention, the FBG and OGTT AUC levels in the model, EA, EA + A, and A groups were significantly higher than those in the normal group (*p* < 0.01). However, no significant difference in FBG was observed among the model, EA, EA + A, and A groups (*p* > 0.05).

After 4 weeks of intervention, the AUC of OGTT in the EA group decreased significantly compared to pre-intervention (*p* < 0.05). The model group exhibited significantly higher FBG, HbA1c, and AUC of OGTT levels than the normal group (*p* < 0.01). In contrast, the EA group showed significantly lower FBG, HbA1c, and AUC of OGTT levels than the model group (*p* < 0.05). Following oral antibiotic treatment of T2DM rats, the hypoglycemic effect of EA attenuated, leading to significant increases in FBG and AUC of OGTT in the EA + A and A groups than the EA group (*p* < 0.05). In addition, the concentration of HbA1c was significantly higher in the A group than in the EA group (*p* < 0.05).

### 3.3 Effect of EA on FINS and HOMA-IR of T2DM rats

**Figure 3:**
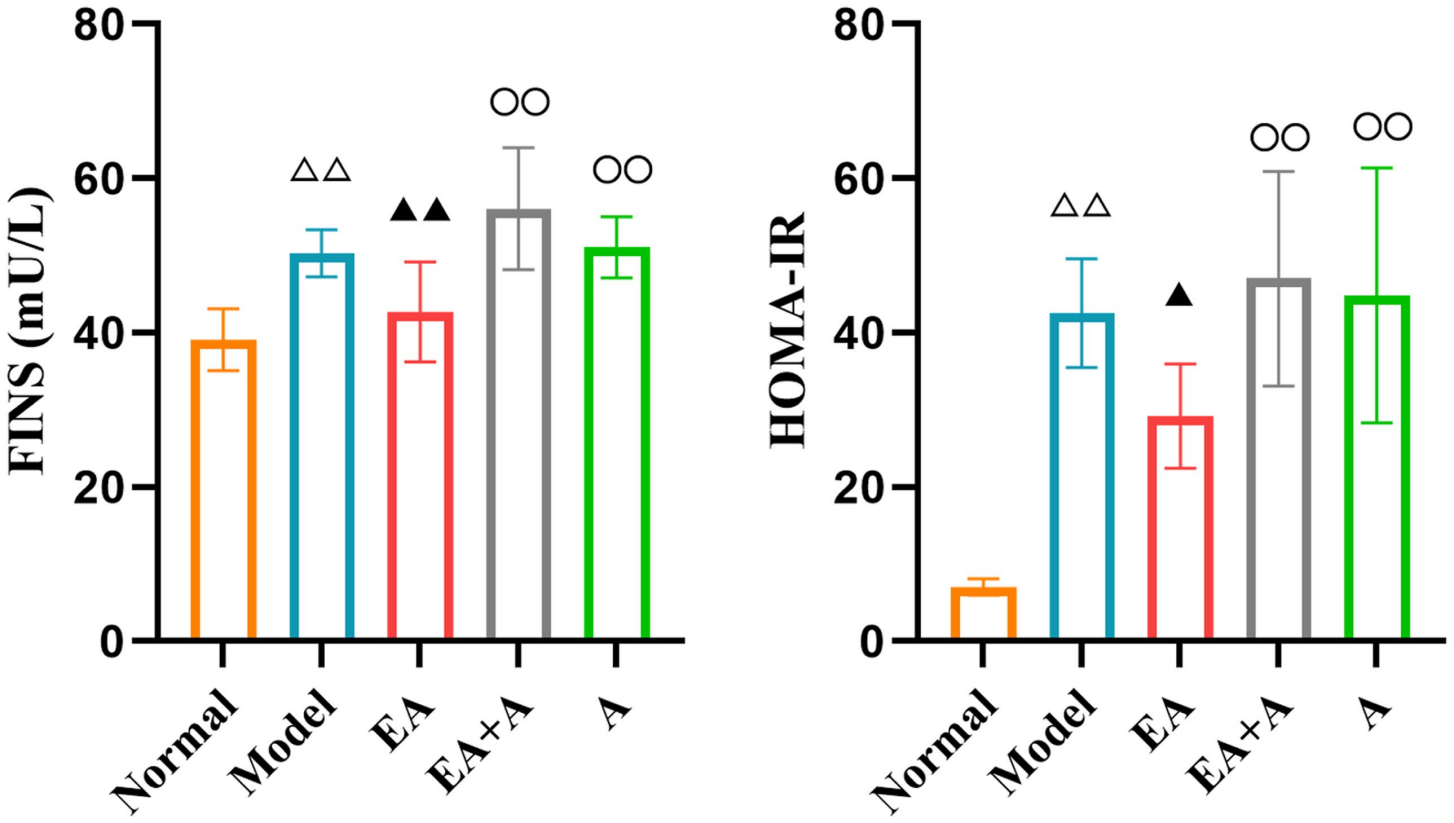
Effect of EA on the FINS and HOMA-IR. ^△△^*p* < 0.01, vs. normal group; ^▲^*p* < 0.05, ^▲▲^*p* < 0.01, vs. model group; ^○○^*p* < 0.01, vs. EA group.

Figure 3 displays the results for FINS and HOMA-IR, which were significantly higher in the model group than the normal group (*p* < 0.01). The EA group exhibited significantly lower FINS and HOMA-IR values than the model group (*p* < 0.05). Following oral antibiotic intervention, both the EA + A and A groups showed significantly higher FINS and HOMA-IR levels than the EA group (*p* < 0.01). These findings suggest that EA may mitigate insulin resistance in T2DM rats, with this therapeutic effect being attenuated by oral antibiotics.

### 3.4 Effect of EA on islet morphology in T2DM rats

**Figure 4:**
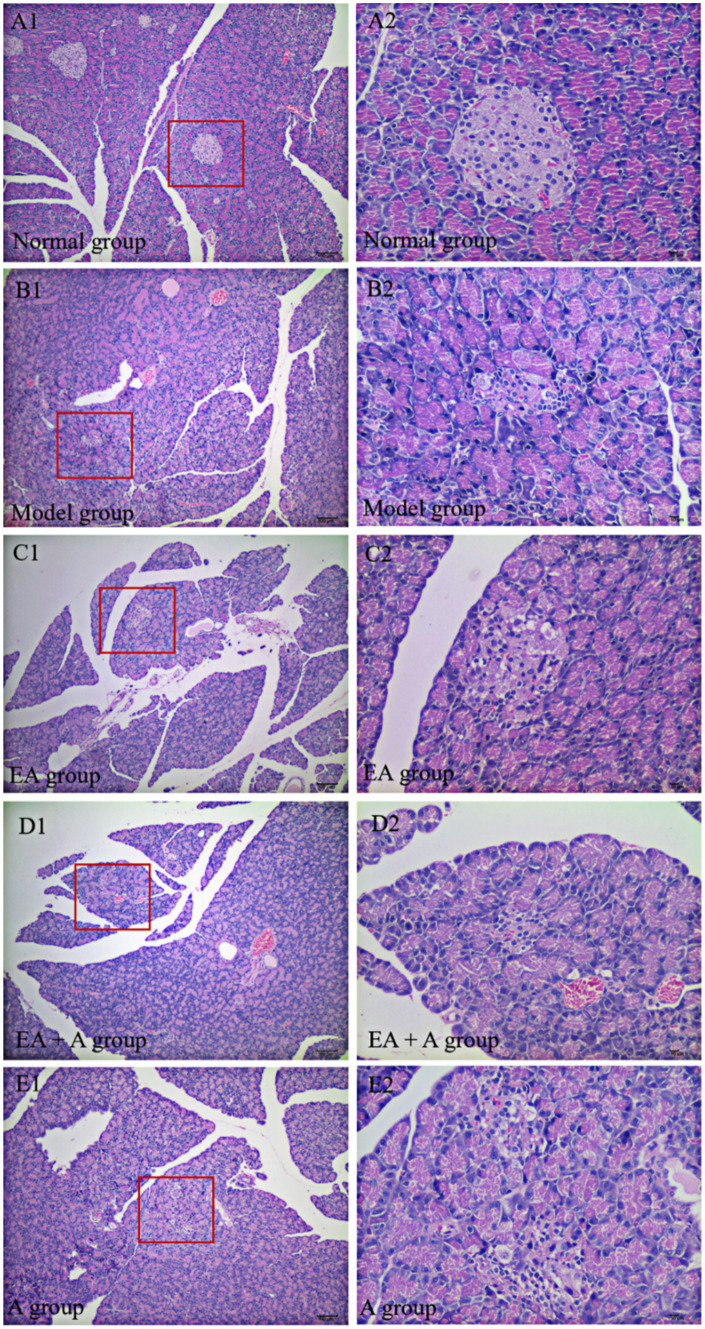
Effect of EA on islet morphology. (**A1,B1,C1,D1,E1**: 100×; **A2,B2,C2,D2,E2**: 400×).

The results of HE staining are presented in Figure 4. In the normal group, pancreatic islets exhibited a complete, oval structure with distinct boundaries. *β*-cells and nuclei within the islets displayed regular morphology and uniform distribution. In contrast, the model group showed irregular islet morphology, reduced islet area, indistinct borders, decreased β-cell nuclei, and compensatory β-cell nuclear enlargement. EA treatment partially alleviated these pathological changes. However, in the EA + A and A groups, pancreatic islet morphology remained irregular, islet area was reduced, and β-cells were disorganized. These findings indicate that EA ameliorate islet damage, and antibiotics reverse this effect.

### 3.5 Effect of EA on the gut microbiota of T2DM rats

#### 3.5.1 Effect of EA on the abundance of OTUs in T2DM rats

**Figure 5:**
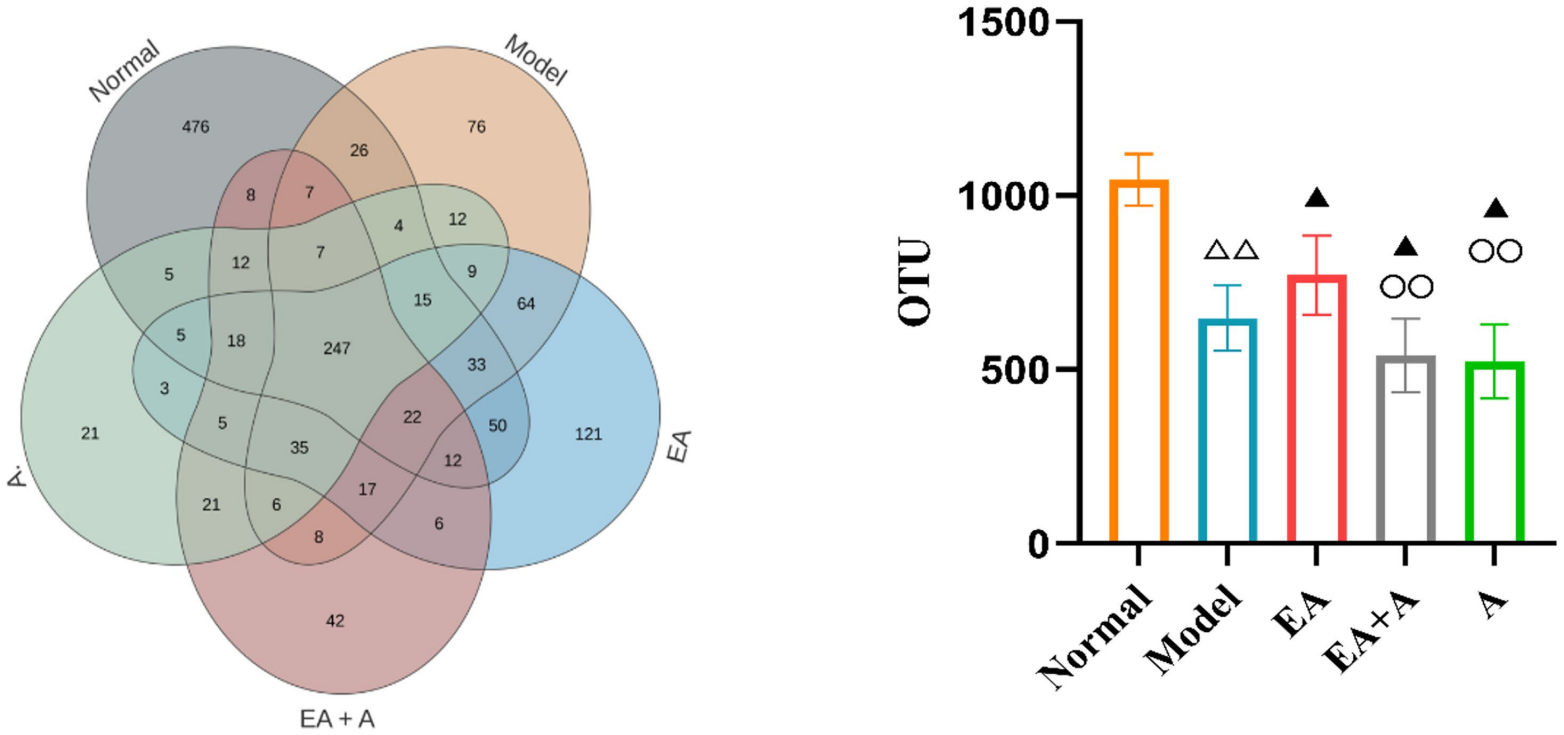
Effect of EA on the number of OTUs. ^△△^*p* < 0.01, vs. normal group; ^▲^*p* < 0.05, vs. model group; ^○○^*p* < 0.01, vs. EA group.

We grouped sequences with ≥97% similarity into OTUs, each representing a distinct microbial species. Prior to intervention, the OTU counts in the model, EA, EA + A, and A groups were significantly lower (*p* < 0.01) than the normal group. Furthermore, the OTU counts in the EA + A and A groups were significantly lower (*p* < 0.05) than those in the model and EA groups. The post-intervention OTU profiles are depicted in Figure 5. The unique OTU counts for the normal, model, EA, EA + A, and A groups were 476, 76, 121, 42, and 21, respectively. The number of shared OTUs among groups was 247, constituting 26.08, 42, 37.3, 52.22, and 58.12% of the total OTUs in the normal (*n* = 947), model (*n* = 588), EA (*n* = 662), EA + A (*n* = 473), and A (*n* = 425) groups, respectively. Compared to the normal group, the model group exhibited a significant decrease in OTU counts (*p* < 0.01). The EA group showed a significant increase in OTU counts compared to the model group (*p* < 0.05). In addition, the OTU counts in the EA + A and A groups were significantly lower than in the EA group (*p* < 0.01). These findings indicate that the intestinal microbiota of normal rats differs from that of T2DM rats, antibiotics reduce OTUs diversity, and EA treatment increases OTUs diversity in T2DM rats.

#### 3.5.2 Effect of EA on alpha diversity and beta diversity of T2DM rats

**Figure 6:**
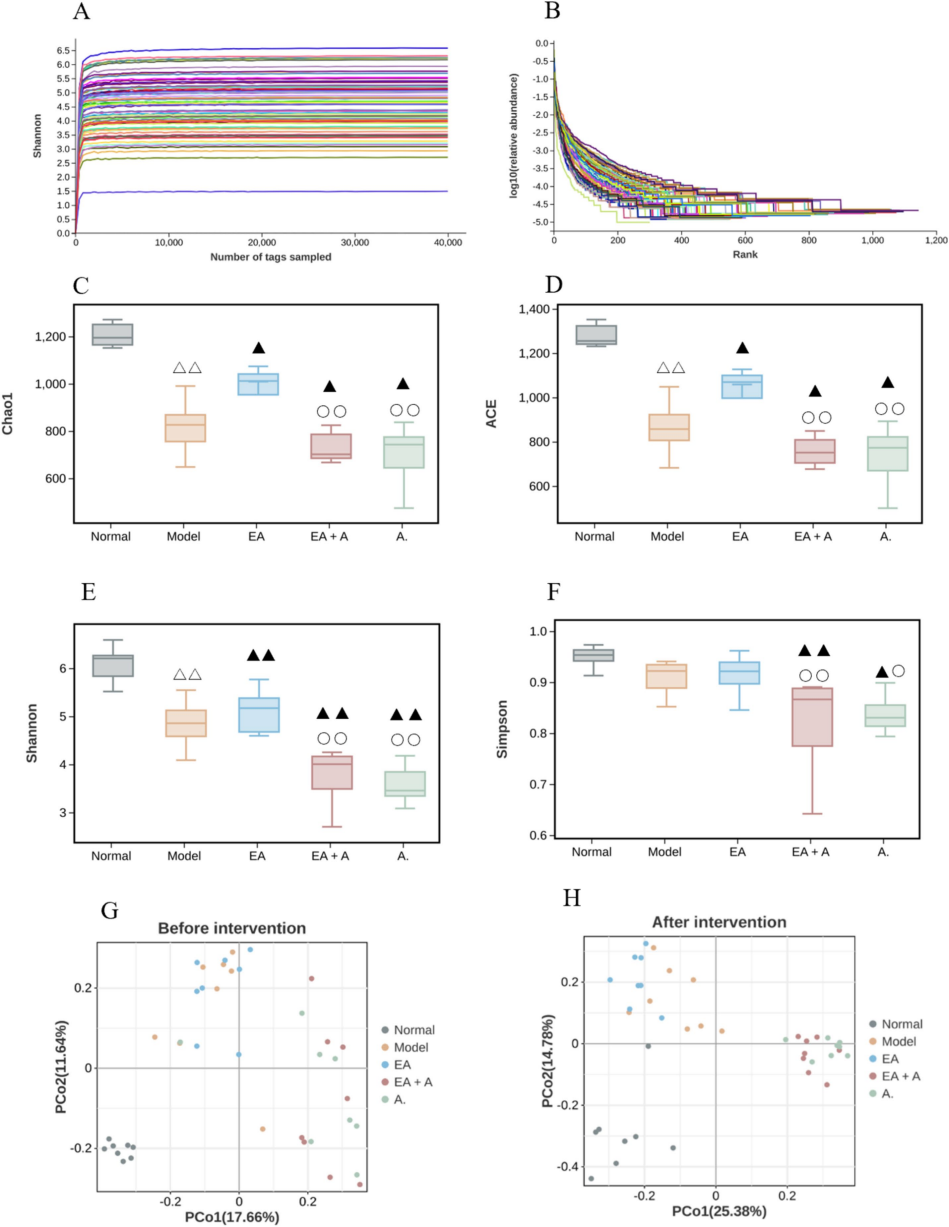
Effect of EA on the alpha diversity and beta diversity. ^△△^*p* < 0.01, vs. normal group; ^▲^*p* < 0.05, ^▲▲^*p* < 0.01, vs. model group; ^○^*p* < 0.05,^○○^*p* < 0.01, vs. EA group. (**A**: Rarefaction curve; **B**: rank abundance; **C**: Chao 1 index; **D**: ACE index; **E**: Shannon index; **F**: Simpson index; **G**: beta diversity before intervention; **H**: beta diversity after intervention).

To assess sequencing depth, sample abundance, and homogeneity, rarefaction and rank abundance curves were employed. As depicted in Figures 6A,B, the plateauing of both curves indicates sufficient sequencing depth and suggests high sample abundance and homogeneity.

Before the intervention, the Shannon, Chao1, and Ace indices were significantly reduced in the model, EA, EA + A, and A groups compared to the normal group (*p* < 0.05), with the Shannon index in the EA + A and A groups being significantly lower than those in the model and EA groups (*p* < 0.05). The analysis of alpha diversity at post-intervention is shown in Figures 6C–F. After the intervention, Shannon, Chao1, and Ace indices decreased significantly in the model group compared to the normal group (*p* < 0.01), while they increased significantly in the EA group compared to the model group (*p* < 0.05). In contrast, the Shannon, Simpson, Chao1, and Ace indices in the EA + A and A groups remained significantly lower than those in the model and EA groups (*p* < 0.05). These findings indicate a decrease in the abundance and diversity of intestinal microorganisms in T2DM rats, an improvement in alpha diversity by EA, and a reduction in alpha diversity by antibiotics.

Figures 6G,H illustrate the results of beta diversity analysis, revealing distinct microbial community structures between the normal and other groups prior to intervention. Post-intervention, the samples from each group formed separate clusters, indicating significant variation in species beta diversity across groups.

#### 3.5.3 Effect of EA on species composition of T2DM rats

**Figure 7:**
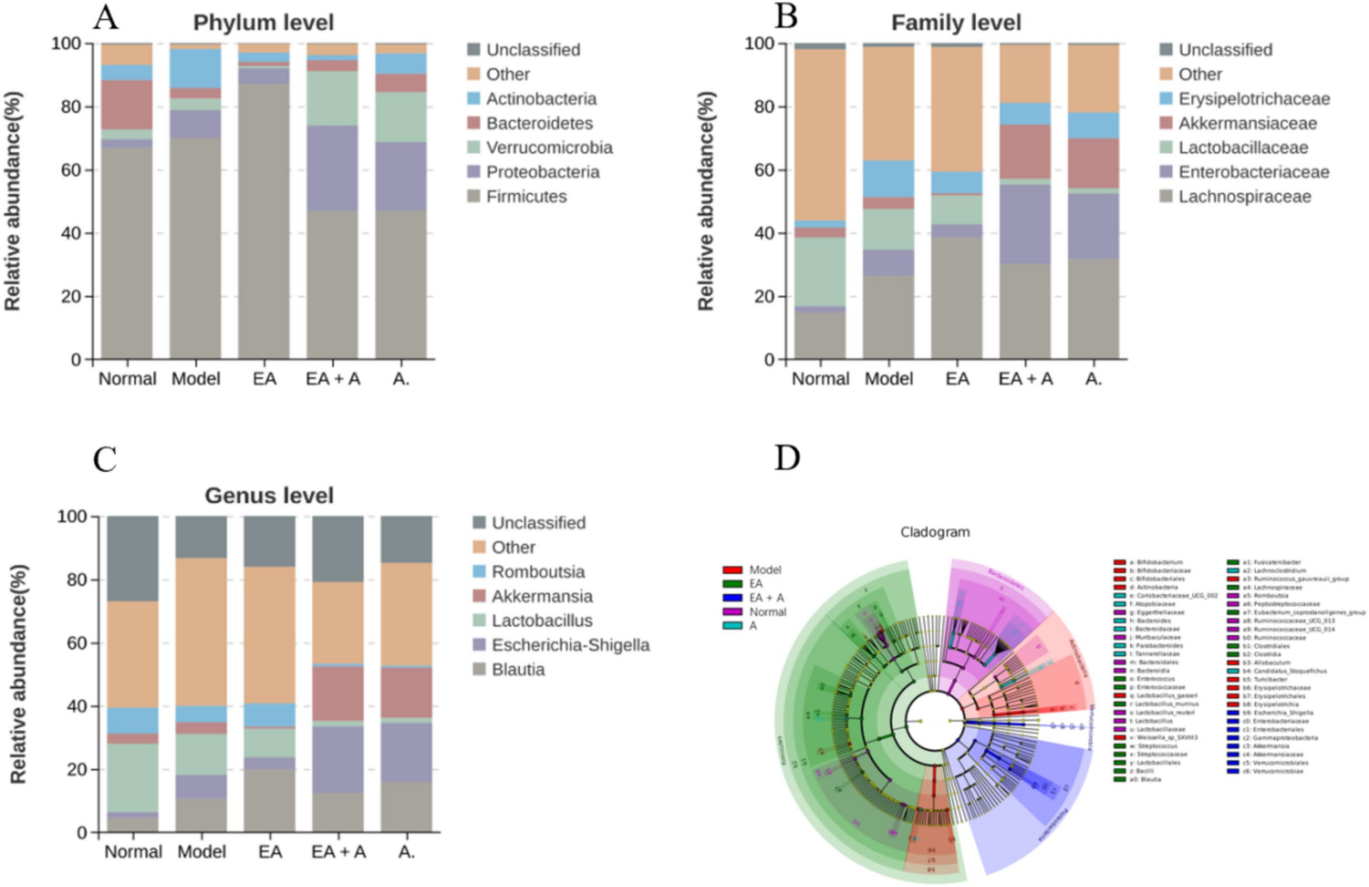
Effect of EA on species composition of T2DM rats. (**A**: top five gut microorganisms at phylum level; **B**: top five gut microorganisms at family level; **C**: top five gut microorganisms at genus level; **D**: the evolutionary branch diagram of LEfSe analysis).

Figures 7A–C illustrate the compositional changes in gut microbiota at the phylum, family, and genus levels. Prior to intervention, the dominant phyla in the normal group were *Firmicutes*, *Bacteroidetes*, and *Proteobacteria*, while in T2DM rats, *Firmicutes*, *Proteobacteria*, and *Verrucomicrobia* predominated. Post-intervention, the model group exhibited a decrease in *Bacteroidetes* and an increase in *Proteobacteria* and *Actinobacteria* compared to the normal group. The EA group displayed a higher proportion of *Firmicutes* and lower proportions of *Actinobacteria* and *Proteobacteria* than the model group. Conversely, the EA + A group showed a decrease in *Firmicutes* and an increase in *Proteobacteria* and *Verrucomicrobia* relative to the EA group. In addition, the EA + A group had a lower proportion of *Bacteroidetes* and a higher proportion of *Proteobacteria* than the A group.

At baseline, *Lachnospiraceae*, *Lactobacillaceae*, and *Peptostreptococcaceae* were dominant in the normal group, while *Lachnospiraceae*, *Lactobacillaceae*, and *Enterobacteriaceae* were dominant in T2DM rats. Post-intervention, the model group exhibited increased *Lachnospiraceae*, *Enterobacteriaceae*, and *Erysipelotrichaceae*, with decreased *Lactobacillaceae* compared to the normal group. The EA group showed further increases in *Lachnospiraceae*, while *Enterobacteriaceae* and *Erysipelotrichaceae* decreased compared to the model group. In the EA + A group, *Lactobacillaceae* decreased, and *Enterobacteriaceae* and *Akkermansiaceae* increased compared to the EA group. In addition, *Enterobacteriaceae* increased in the EA + A group relative to the A group.

At the genus level, prior to intervention, *Lactobacillus*, *Romboutsia*, and *Blautia* were dominant in the normal group, while *Blautia*, *Lactobacillus*, and *Escherichia–Shigella* predominated in T2DM rats. Post-intervention, the model group exhibited decreased *Lactobacillus* and *Romboutsia*, along with increased *Blautia* and *Escherichia–Shigella* compared to the normal group. Conversely, the EA group showed increased *Blautia* and *Romboutsia* and decreased *Escherichia–Shigella* relative to the model group. In contrast to the EA group, the EA + A group displayed decreased *Blautia*, *Lactobacillus*, and *Romboutsia* and increased *Escherichia–Shigella* and *Akkermansia*. Furthermore, *Escherichia–Shigella* was more abundant in the EA + A group than in the A group. These findings indicate that EA intervention has positively influence the gut microbiota composition in T2DM rats.

As presented in Figures 7D, 53 characteristic flora changed significantly (LDA score > 4, *p* < 0.05). In T2DM rats, dominant bacterial groups included *Actinobacteria*, *Erysipelotrichaceae*, *Bifidobacterium*, *Lactobacillus_gasseri*, *Turicibacter*, *Allobaculum*, *Ruminococcus_gauvreauii_group*, *and Weissella_sp_SXVIII3*. EA treatment increased the abundance of *Firmicutes*, *Lachnospiraceae*, *Bacilli*, *Lactobacillales*, *Clostridia*, *Clostridiales*, and *Blautia* while decreasing *Actinobacteria*, *Erysipelotrichaceae*, *Bifidobacterium*, *Lactobacillus*_gasseri, *Turicibacter*, and *Allobaculum*. In the EA group, dominant bacteria included *Firmicutes*, *Lachnospiraceae*, *Bacilli*, *Lactobacillales*, *Clostridia*, *Clostridiales*, *Blautia*, *Streptococcus*, *Lactobacillus_murinus*, *Eubacterium_coprostanoligenes_group*, *Enterococcus*, and *Fusicatenibacter*. The EA + A group was dominated by *Gammaproteobacteria*, *Proteobacteria*, *Enterobacteriales*, *Escherichia_Shigella*, *Verrucomicrobiales*, and *Akkermansia*. In the A group, *Candidatus_Stoquefichus*, *Atopobiaceae*, *Coribacteriaceae-UCG-002*, *Lachnoclostridium*, *Tannerellaceae*, *Parabacteroides*, and *Bacteroidaceae* were dominant.

### 3.6 Correlation analysis between gut microbiota and glucose metabolism-related indicators

The correlation analysis revealed significant associations between specific gut microbiota and glucose metabolism-related indicators (FBG, OGTT, FINS, HOMA-IR, and HbA1c). *Lactobacillaceae* and *Lactobacillus* exhibited negative correlations with FBG (*R* = −0.81, *p* = 0.015 and *R* = −0.759, *p* = 0.029, respectively). Conversely, *Peptostreptococcaceae* and *Romboutsia* were negatively associated with HbA1c (*R* = −0.81, *p* = 0.015), while *Enterobacteriaceae* showed a positive correlation with the OGTT results (*R* = 0.762, *p* = 0.028).

### 3.7 Effect of EA on SCFAs in T2DM rats

**Figure 8:**
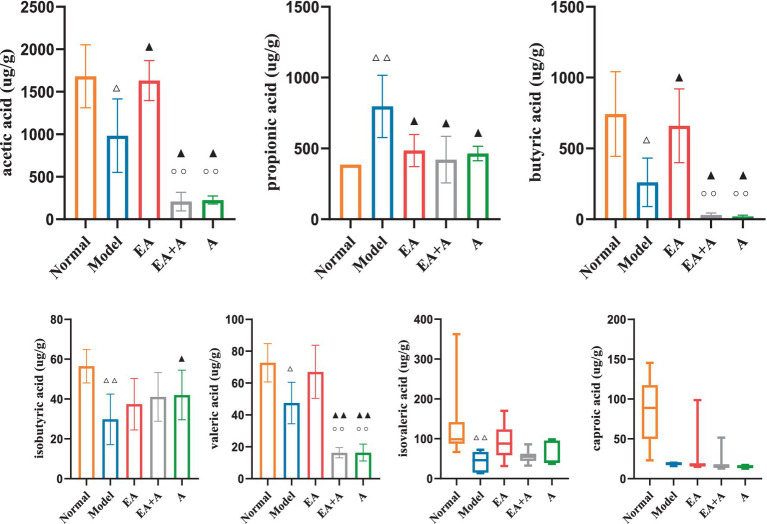
Effect of EA on the SCFAs. ^△^*p* < 0.05, ^△△^*p* < 0.01, vs. normal group; ^▲^*p* < 0.05, ^▲▲^*p* < 0.01, vs. model group; ^○○^*p* < 0.01, vs. EA group.

Figure 8 presents the SCFA analysis results. The model group exhibited decreased levels of acetic acid, butyric acid, isobutyric acid, valeric acid, and isovaleric acid compared to the normal group, while propionic acid levels increased (*p* < 0.05). In contrast, the EA group showed elevated acetic acid and butyric acid concentrations and reduced propionic acid levels (*p* < 0.05) compared to the model group. Interestingly, the EA + A and A groups displayed decreased acetic acid, butyric acid, and valeric acid levels (*p* < 0.05) relative to the EA group. These findings suggest that EA upregulates SCFA content, while antibiotic administration downregulates it.

### 3.8 Effect of EA on GLP-1 of T2DM rats

**Figure 9:**
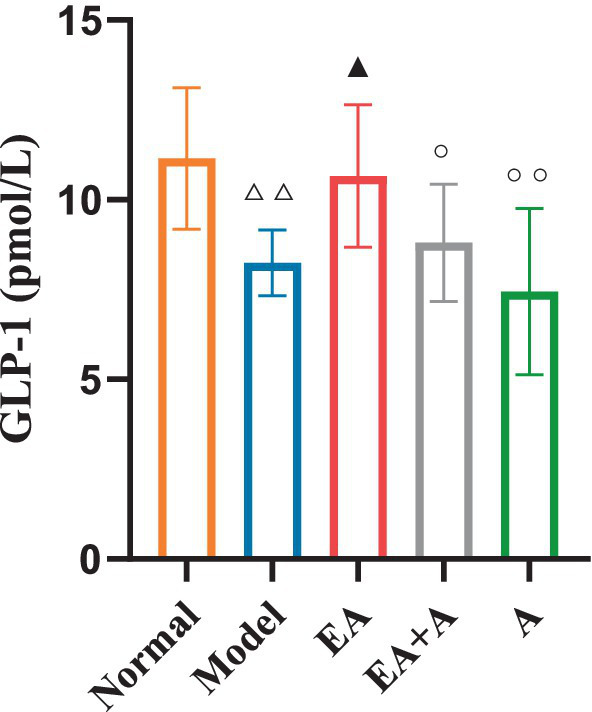
Effect of EA on the GLP-1. ^△△^*p* < 0.01, vs. normal group; ^▲^*p* < 0.05, vs. model group; ^○^*p* < 0.05, ^○○^*p* < 0.01, vs. EA group.

Figure 9 illustrates the impact of GLP-1 on the studied groups. The model group exhibited significantly lower GLP-1 levels than the normal group (*p* < 0.01). While the EA group showed a significant increase in GLP-1 concentration relative to the model group (*p* < 0.05), the administration of antibiotics attenuated this effect.

### 3.9 Correlation analysis between GLP-1 and SCFAs

**Figure 10:**
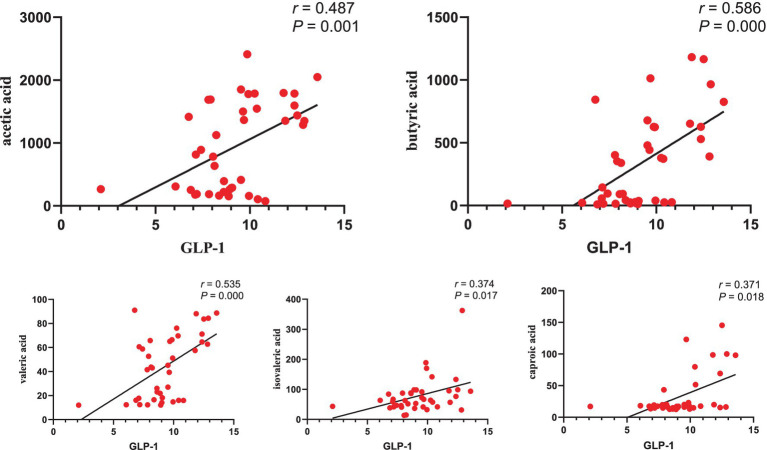
Correlation analysis between GLP-1 and SCFAs.

The correlation analysis (Figure 10) revealed a positive association between GLP-1 levels and the concentrations of acetic acid (*R* = 0.487, *p* = 0.001), butyric acid (*R* = 0.586, *p* = 0.000), valeric acid (*R* = 0.535, *p* = 0.000), isovaleric acid (*R* = 0.374, *p* = 0.017), and caproic acid (*R* = 0.371, *p* = 0.018).

## 4 Discussion

### 4.1 EA could reduce blood glucose, ameliorate islet damage, and improve the symptoms of T2DM rats

The present study revealed that EA treatment significantly improved blood glucose levels and IR in rats with T2DM. Specifically, the EA group exhibited lower levels of FBG, HbA1c, FINS, AUC of OGTT, and HOMA-IR than the model group. These findings aligned with previous studies demonstrating the beneficial effects of EA on glycemic metabolism in T2DM. (Zhang et al., 2021). Xu et al. (2022) reported that EA reduced FBG and improved glucose tolerance. In addition, research has shown that EA at the “Weiwanxiashu”(EX B3) acupoint could effectively lower FBG levels by regulating pancreatic function and increasing the expression of the glucagon-like peptide-1 receptor (Cao et al., 2017). Furthermore, EA treatment has been observed to restore the overall morphology and area of pancreatic islets, increase the number of *β*-cell nuclei, and alleviate compensatory β-cell enlargement (Cao et al., 2017). Notably, our study also revealed that EA alleviated the symptoms of excessive drinking, excessive eating, and weight gain in T2DM rats. Ting et al. (2022) observed that EA ameliorated the symptoms of polydipsia and polyphagia, rough and lusterless hair, and urination in diabetic mice. Xu et al. (2016) reported that EA could effectively reduce blood glucose and FINS levels, improve insulin sensitivity and insulin resistance, and maintain the body mass of diabetic rats.

In summary, EA had the potential to improve glucose metabolism, as evidenced by reductions in FBG and HbA1c levels. In addition, EA appeared to alleviate insulin resistance, as indicated by the lower levels of FINS, AUC of OGTT, and HOMA-IR. Furthermore, EA treatment was associated with amelioration of islet damage and related symptoms such as polydipsia, polyphagia, and weight loss in T2DM rats.

### 4.2 The hypoglycemic effect of EA was associated with its regulation of the gut microbiota

The intestinal microbiota plays a crucial role in various physiological processes, including immune function enhancement, food digestion, intestinal endocrine regulation, metabolic modulation, and toxin elimination (Sanna et al., 2019). Accumulating evidence suggests a strong association between the intestinal microbiota and the development and progression of T2DM (Sanna et al., 2019; Zhernakova et al., 2016). In the present study, the number of OTUs and the indices of Chao1, ACE, Shannon, and Simpson decreased in the model group compared to the normal group. This finding aligned with previous research indicating a reduction in gut microbiota diversity in individuals with T2DM (Gong et al., 2021; Wang et al., 2020). Meanwhile, in contrast to the normal group, the proportion of *Proteobacteria*, *Actinobacteria*, *Lachnospiraceae*, *Enterobacteriaceae*, *Erysipelotrichaceae*, *Blautia*, and *Escherichia–Shigella* increased, while the proportion of *Bacteroidetes*, *Lactobacillaceae*, *Lactobacillus*, and *Romboutsia* decreased in the T2DM rats. The increased abundance of *Actinobacteria* in the T2DM group could potentially trigger increased intestinal permeability, leading to bacterial translocation into the bloodstream and tissues and subsequently contributing to IR and glucose metabolic disorders (Liu et al., 2023; Xiang et al., 2020). *Allobaculum* produces trimethylamine oxide (TMAO) (Zhu et al., 2016), which may promote fat production by inhibiting bile acid-mediated hepatic farnesoid X receptor signaling, inducing IR, thereby affecting blood glucose homeostasis and promoting the development of T2DM (Chen et al., 2022). Zhou et al. (2019) reported a positive correlation between *Allobaculum* abundance and random blood glucose levels in T2DM rats. *Proteobacteria* can generate large amounts of lipopolysaccharides, which contribute to diabetes by increasing pro-inflammatory cytokine levels and impairing pancreatic *β*-cell function (Wu et al., 2021; Zhang et al., 2022). *Escherichia–Shigella* and *Enterobacteriales* are Gram-negative bacteria, which are associated with inflammation, disruption of the intestinal barrier, and subsequent insulin resistance, hyperglycemia, and T2DM (Pinaud et al., 2018; Huang et al., 2021; Pedersen et al., 2018). In the present study, *Enterobacteriaceae* abundance was positively correlated with OGTT. Several studies have shown that antibiotic administration can reduce the abundance of fecal microbiota, disrupt the gut microbiota structure, and lead to microbial depletion (Josefsdottir et al., 2017; Liang et al., 2020). In our study, gut microbiota diversity decreased in both the EA + A and A groups, demonstrating a successful establishment of pseudo-sterile model. After antibiotic intervention, the therapeutic effects of EA on polyphagia, polydipsia, weight loss, hyperglycemia, glucose intolerance, insulin resistance, and dyslipidemia in T2DM rats were reversed, indicating that antibiotics can attenuate the beneficial effects of EA in T2DM.

Studies have documented that certain pathogenic bacteria, such as *Proteobacteria*, *Actinobacteria* (Rehman et al., 2022), *Escherichia–Shigella* (Deng et al., 2022), *Enterobacteriales* (Du et al., 2021), and *Allobaculum* (Yanling et al., 2022), are involved in the development of T2DM. EA can effectively improve intestinal microbial diversity, increase beneficial bacteria, reduce harmful bacteria, restore intestinal microecological balance, and improve disease conditions (Wang et al., 2022; Li et al., 2023). In the present study, EA increased microbial diversity and species richness and affected the microbial structure in T2DM rats. Pan et al. (2023) and An et al. (2022) reported that EA increased the Shannon index in T2DM rats. In addition, EA increased *Firmicutes*, *Lachnospiraceae*, *Blautia,* and *Romboutsia* while decreasing *Actinobacteria*, *Proteobacteria*, *Enterobacteriaceae*, *Erysipelotrichaceae*, and *Escherichia–Shigella* in T2DM rats. LEfSe analysis revealed that the dominant flora in T2DM rats included *Actinobacteria*, *Erysipelotrichaceae*, *Bifidobacterium*, *Lactobacillus_gasseri*, *Turicibacter*, and *Allobaculum*. In contrast, the dominant flora of rats after EA treatment included *Firmicutes*, *Lachnospiraceae*, *Bacilli*, *Lactobacillales*, *Clostridia*, *Clostridiales*, *Blautia*, and others. Chen et al. reported that Romboutsia could utilize glucose to produce butyric acid and acetic acid, which are beneficial for obesity (Chen et al., 2022). Butyric acid-producing bacteria, such as *Peptostreptococcaceae*, are inversely associated with insulin resistance or T2DM (Chen et al., 2021). Moreover, our findings demonstrated that *Peptostreptococcaceae* and *Romboutsia* were negatively correlated with HbA1c, which played a beneficial regulatory role in glucose metabolism for T2DM. Evidence suggests that Bacilli and *Clostridiales* can inhibit NF-κB activity, reduce the expression of pro-inflammatory factors, and exert anti-inflammatory effects (Zixin, 2019; Cunningham et al., 2021). Beneficial bacteria, such as *Lactobacillales* and *Lactobacillus*, can affect glucose absorption, regulate lipid metabolism, inhibit inflammation, reduce LPS content, and exert hypoglycemic and hypolipidemic effects (Tonucci et al., 2017; Ng et al., 2022; Chai et al., 2021). In the present study, *Lactobacillaceae* and *Lactobacillus* were negatively correlated with FBG. *Lachnospiraceae* can produce SCFAs, which are involved in the metabolism of multiple carbohydrates and fats (Chen et al., 2021; Zhang et al., 2021). SCFAs can enhance the acidic environment in the intestinal tract, inhibit the growth of harmful bacteria, and alleviate mucosal inflammation. In addition, SCFAs can combine with G protein-coupled receptors to exert hypoglycemic functions (Ma et al., 2019). The composition of the gut microbiota in the EA + A and A groups was similar to that of the T2DM group, indicating that both T2DM and long-term oral antibiotics can disrupt the gut microbiota structure. In summary, gut microbiota is closely related to T2DM, and EA can improve T2DM glucose metabolism by regulating gut microbiota. Gut microbiota plays an important role in the therapeutic effect of EA.

### 4.3 EA could regulate the production of SCFAs and stimulate the secretion of GLP-1, thereby exerting a hypoglycemic effect

The alteration of SCFAs caused by dysbiosis is a crucial mechanism underlying the involvement of gut microbiota in the pathogenesis of T2DM. Serena et al. discovered that an increase in intestinal production of the SCFA butyrate was linked to improved insulin response after oral glucose testing, while propionate was causally associated with an increased risk of T2DM (Sanna et al., 2019). Several studies have found that the abundance of butyrate-producing bacteria (such as *Roseburia, Subdoligranulum, Ruminococcus,* and *Clostridiales* spp.) in the intestines of patients with T2DM decreased, leading to increased abundance of pathogenic bacteria and subsequent insulin resistance and elevated blood glucose (Cunningham et al., 2021; Forslund et al., 2015). Meanwhile, we found that the levels of acetic acid, butyric acid, isobutyric acid, valeric acid, and isovaleric acid decreased, while propionic acid increased in T2DM rats. In contrast, EA increased the concentration of acetic acid and butyric acid and decreased the concentration of propionic acid. Patients with T2DM (An et al., 2023) and T2DM rats (Wang et al., 2022) exhibited lower acetic and butyric acid levels. Studies have documented that SCFAs, such as butyric acid, could increase the release of GLP-1, forming a feedback pathway for metabolic homeostasis, thereby lowering blood glucose and improving glucose metabolism (Tolhurst et al., 2012; Mazhar et al., 2023). Our study showed that GLP-1 was positively correlated with acetic acid (Cao et al., 2017), butyric acid, isovaleric acid, valeric acid, and caproic acid. Cao et al. found that EA could increase pancreatic GLP-1 receptor expression, partially restore islet morphology, and contribute to lowering blood glucose. Similarly, growing evidence suggests that EA can remodel the structure of the gut microbiota, increase intestinal SCFAs, affect circulating LPS levels, and reduce inflammatory responses (Zhang et al., 2021; An et al., 2022). In contrast to previous studies, we conducted in-depth research on the downstream effector GLP-1 of SCFAs. We also established pseudo-sterile rats to reversely verify that EA could regulate gut microbiota and SCFAs, affect GLP-1 secretion and lower blood glucose.

### 4.4 Limitations and implication for future studies

There are several limitations in the present study. First, our findings suggest that EA improves glucose metabolism in T2DM rats by modulating gut microbiota and SCFAs, while the specific role of individual bacteria in the therapeutic effect of EA has not been directly investigated. Techniques such as gut microbiota gene knockout, FMT, and metagenomic sequencing analysis could be employed to further identify specific bacteria or microbial products involved in the hypoglycemic efficacy of EA. Second, a sham EA group was not set as a comparison. A group without electrical stimulation or a sham acupoint group could serve as a control to elucidate the specific effects of EA. Third, antibiotics were used to deplete gut microbiota as a pseudo-sterile model. Although this approach reduced the majority of bacterial species, some bacteria may persist in the gut. A germ-free model could be used to eliminate the influence of gut microbiota, allowing for a direct validation of the relationship between the hypoglycemic efficacy of EA and gut microbiota. Fourth, we detected changes in gut microbiota and islet morphology and preliminarily investigated the hypoglycemic mechanism of EA, the existing evidence demonstrates a strong association between gut microbiota and impaired gut barrier function and inflammation during the progression of T2DM (Yao et al., 2022; Gurung et al., 2020). However, the specific regulatory mechanism of EA on gut microbiota-induced gut barrier damage or immune response remains unclear and warrants further exploration.

## 5 Conclusion

Our findings suggested that EA could improve glucose metabolism in T2DM rats. Its hypoglycemic effect was associated with the regulation of gut microbiota, SCFAs, and GLP-1.

## Data Availability

The authors acknowledge that the data presented in this study must be deposited and made publicly available in an acceptable repository, prior to publication. Frontiers cannot accept a manuscript that does not adhere to our open data policies.

## Footnotes

^1^ https://www.omicsmart.com/
